# Elevated functional magnetic resonance imaging activity in cognitively normal participants predicts future dementia

**DOI:** 10.1093/braincomms/fcag167

**Published:** 2026-05-25

**Authors:** Sabrina G Clemens, Diana A Hobbs, Nicole S McKay, Peter R Millar, John C Morris, Jason Hassenstab, Jeffrey M Zacks, David A Balota, Brian A Gordon

**Affiliations:** Division of Biology & Biomedical Sciences, Washington University in St.Louis, St. Louis, MO, USA; Department of Neurology, Washington University in St.Louis, St. Louis, MO, USA; Department of Radiology, Washington University in St.Louis, St. Louis, MO, USA; Department of Radiology, Washington University in St.Louis, St. Louis, MO, USA; Department of Neurology, Washington University in St.Louis, St. Louis, MO, USA; Department of Neurology, Washington University in St.Louis, St. Louis, MO, USA; Department of Neurology, Washington University in St.Louis, St. Louis, MO, USA; Department of Psychological & Brain Sciences, Washington University in St.Louis, St. Louis, MO, USA; Department of Radiology, Washington University in St.Louis, St. Louis, MO, USA; Department of Psychological & Brain Sciences, Washington University in St.Louis, St. Louis, MO, USA; Department of Psychological & Brain Sciences, Washington University in St.Louis, St. Louis, MO, USA; Department of Radiology, Washington University in St.Louis, St. Louis, MO, USA; Department of Psychological & Brain Sciences, Washington University in St.Louis, St. Louis, MO, USA

**Keywords:** dementia, Alzheimer, fMRI, cognition, ageing

## Abstract

As individuals age, they are more likely to show increased functional MRI (fMRI) activity, particularly in frontal regions. This has been interpreted as a compensatory mechanism, yet the very need to draw upon such resources indicates increasing failures of brain systems. Almost all work addressing theoretical models explaining these patterns has been cross-sectional, with minimal work testing how elevated fMRI activity predicts future cognitive trajectories. Further, although often viewed as ageing, there is increasing evidence that subtle fMRI changes may represent the earliest manifestation of neurodegenerative conditions such as Alzheimer’s disease. One hundred nine individuals completed a Stroop colour/word task during fMRI data acquisition. Analyses focused on differences between trials where the colour and word were incongruent (the word red written in blue) relative to congruent trials (the word blue written in blue). At baseline, participants also underwent amyloid positron emission tomography imaging and *APOE* genotyping. Individuals had longitudinal clinical follow-up (mean 6.8 years) with 15 individuals reaching the threshold of clinically defined cognitive impairment. Across the entire cohort, several brain regions, including dorsolateral prefrontal cortex, anterior cingulate cortex, and lateral temporal and parietal regions, were more active on conflict trials. At the individual level, increases in activity were related to changes in reaction time, with those experiencing greater conflict having greater evoked activity. Further, individuals who later developed dementia had greater activity at baseline than their peers who remained cognitively normal despite there being no differences in accuracy (*t* = 0.23, *P* = 0.82) or reaction time (*t* = 0.94, *P* = 0.35) in the Stroop task nor differences in mini-mental state examination (*t* = 0.06, *P* = 0.95) or a neuropsychological composite (*t* = −0.99, *P* = 0.32). Individuals who progressed were more likely to be amyloid positive (*χ*^2^ = 26.71, *P* = 0.000002) and carriers of the *APOE* ε4 allele (*χ*^2^ = 4.81, *P* = 0.03). The current work suggests that, although compensatory in nature in the short term, increased activation of frontal and parietal control regions during attentional control tasks is indicative of underlying declines in brain health. The larger implications are 2-fold. Undetected neurodegenerative disease pathology biases our understanding of what constitutes healthy ageing. Further, alterations in brain function occur well in advance of clinically detectable cognitive change, emphasizing the need to intervene with disease modifying therapies early in the disease course.

## Teaser

In the current study, Clemens *et al*. tested whether brain activity while performing a demanding cognitive task predicted later cognitive decline. They found that individuals that developed dementia had greater brain activity at baseline despite no outward impairment. This suggests that, although perhaps initially compensatory, hyperactivation represents declining brain health.

## Graphical Abstract

**Graphical Abstract:**
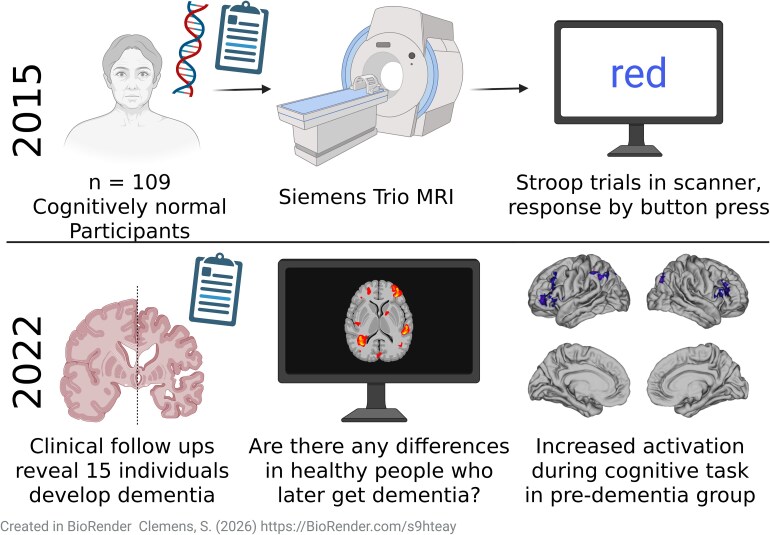

## Introduction

By 2050, the proportion of the global population over the age of 60 is projected to rise more than 20%.^1^ This shift in demographic composition will have reverberating effects on society, affecting healthcare systems, social policies and many sectors of the economy. While treating age-related illnesses like cardiovascular disease and cancer^2^ have tremendously extended the lifespan, the ultimate societal goal now is to improve the quality of life in later years. To achieve this, it is crucial that scientific research prioritizes understanding the impact of ageing—and age-related diseases—on the brain and cognition.

Functional MRI (fMRI), one of the most commonly utilized measures to study the brain, leverages the blood oxygen level-dependent (BOLD) response as an indirect measure of neural activity. fMRI has been widely used to characterize activation differences between older and younger adults during cognitive task performance. One often discussed finding is that older adults can exhibit greater task-related activity than younger adults.^3-19^ This typically appears as an increased magnitude of activation within areas engaged by younger adults, activation of additional areas, and reduced suppression of competing networks such as the default mode network (DMN). The recruitment of additional areas by older adults primarily occurs in frontal regions, which has been recognized in the field as a posterior to anterior shift in the focus of brain activity.^4,20^ Furthermore, activity in older adults shows reduced hemispheric asymmetry compared to younger adults.^4,21,22^ While the more commonly observed pattern is greater activity in older adults, some findings report reduced activity with advancing age.^13,15,19,20,23-27^ Reduced activity is most common in temporal lobe structures during memory paradigms and can occur in the presence of increased activity in other networks. As a result, it may represent a downstream or parallel effect to increases in attentional control networks.

Why older adults show altered activity relative to younger adults during cognitive tasks has been a major focus in the field over the last decade. Prior work has shown that greater task-evoked activity in older adults is associated with both improved^15,17,18,20,21,28,29^ and diminished performance on cognitive tasks.^11,13,18,30^ A more nuanced pattern emerges when task difficulty is varied, with older adults showing increased activity until a difficulty threshold is reached, after which both performance and activity decline.^10,31,32^ The scaffolding theory of ageing and cognition proposes to explain these increased activations as an obstructed compensatory response to age-related neural challenges.^33,34^ While increased activity is beneficial in certain circumstances,^20,21,33,34^ it is not a sustainable or efficient long-term strategy for supporting long-term brain function. Eventually, there will be no more resource to recruit, and behaviour will decline.^31,32,35^ Although more commonly seen in older adults, younger adults also exhibit similar patterns of increased bilateral activations and recruitment of attentional control networks^31,36-38^ under high attentional demands, mimicking results seen in older adults. This suggests that such patterns reflect broader brain organization and resource utilization and are not just a unique feature of ageing.

While the mobilization of additional brain areas is beneficial to meet immediate task demands, the fact that the system requires more resources represents an underlying vulnerability. This likely reflects a degree of neurodegeneration and decline in the brain. If this is the case, then greater functional activity will ultimately be predictive of worse future cognitive trajectories and later risk for developing neurodegenerative disorders such as Alzheimer’s disease . Further, if overactivations of frontal networks are associated with the later onset of dementia, this would suggest it is a feature of pathological rather than healthy ageing. Prior work has demonstrated that the presence of preclinical Alzheimer's disease has been shown to bias age-related estimates of cognition,^39,40^ resting-state fMRI^41^ and structural MRI.^42^ However, fMRI studies of ageing and brain activity are overwhelmingly cross-sectional with a paucity of studies linking brain activity at baseline to subsequent longitudinal cognitive trajectories.^43^ As a result, there is a crucial gap in the literature that is unresolved as to the ultimate implications of overactivations during task performance. To address this question, we analysed fMRI data from cognitively normal older adults performing an attentionally demanding cognitive task who then had longitudinal cognitive evaluations. We focused on whether those individuals who later developed dementia exhibited different activity patterns at baseline compared to their unimpaired peers.

## Materials and methods

### Participants

Participants were enrolled in the Adult Children Study (ACS) at the Charles F. & Joanne Knight Alzheimer Disease Research Center at Washington University in St. Louis. The ACS is enriched for individuals that have at least one parent diagnosed with Alzheimer's disease, placing them at a higher risk for developing Alzheimer's disease dementia. All participants were right-handed, cognitively normal at baseline (Clinical Dementia Rating® [CDR®] = 0^44^) and underwent fMRI with a previously described scanning protocol.^45-48^ After excluding individuals with baseline cognitive impairment (CDR > 0), high motion during fMRI scans (*n* = 23), previous neurological injury (e.g. stroke) and non-native English speaker status, a sample of 120 individuals was identified. This was further refined to 109 based on the availability of follow-up clinical assessments (avg 6.8 years ± 1.9). In the final sample, 94 participants remained cognitively stable while 15 participants had progressed to a cognitively impaired state (CDR > 0). The cohort was largely white (102/109) and female (66/109). The Washington University Human Research Protection Office (HRPO) approved all procedures and participants provided written informed consent in accordance with the Declaration of Helsinki.

### Assessment of Alzheimer’s disease risk at baseline

All participants underwent apolipoprotein (*APOE*) genotyping and were classified based on the presence of an APOE ε4 allele. All participants had an amyloid (Aβ) positron emission tomography (PET) scan within 3 years of their baseline visit (mean = 63.9 days, median = 0, range 0 to 1063 days) with 102/109 (94%) individuals scanned within one year. Pathology was quantified using Pittsburgh Compound B (PiB) PET and scans were processed using FreeSurfer derived regions of interest as previously reported.^49,50^ Standardized uptake value ratios (SUVRs) were calculated in the 30–60 post-injection window relative to a cerebellar grey reference region. Partial volume correction was applied using a regional spread function approach, and global amyloid deposition was defined as the mean SUVR across several cortical regions (precuneus, superior frontal, rostral middle frontal, lateral orbitofrontal, medial orbitofrontal, superior temporal and middle temporal cortices). Participants were classified as Aβ+ using a previously established threshold of 1.42 SUVRs. Cognition was assessed using a previously published preclinical Alzheimer Disease cognitive composite (PACC,^51^), which includes the free recall score from the Free and Cued Selective Reminding Task,^52^ the total correct score from the Digit Symbol subtest of the WAIS-R,^53^ the total completion time from the Trail Making Test Part B^54^ and the total correct score from the Animal Naming Test.^55^ All participants had completed the mini-mental state examinations (MMSE).

*χ*² tests were used to assess the differences in sex, *APOE* genotype and Aβ+ frequency between those who participants who later developed dementia and those who remained cognitively normal. Two sample *t*-tests compared ages between groups. Linear regressions, controlling for age, tested whether individuals who later became demented had higher baseline levels of Aβ and worse baseline MMSE and Knight PACC scores compared to those who remained cognitively normal. Linear mixed effects models, controlling for age, tested whether group differences in BOLD Stroop activity predicted cross-sectional and longitudinal change in Knight PACC scores. Analyses were done using R version 4.4.0.

### Stroop task

See Gordon *et al*.^45^ for a visual depiction of the task. After a brief introduction to the Stroop colour-naming test, participants performed two 9 min and 50 s runs of Stroop trials in the scanner. They responded by selecting the font colour of a presented word (i.e. red or blue), by button press. Stimuli included three conditions: congruent (e.g. ‘blue’ in blue font), incongruent (e.g. ‘red’ in blue font) or neutral (e.g. ‘deep’ in blue font). Each run consisted of four 110-s task blocks interspersed with five 30-s rest intervals. Each block included 24 trials equally distributed across the three Stroop conditions. Trials were presented above a white fixation cross while rest intervals were indicated by a red fixation cross. Each trial was presented on the screen for 1 s followed by an intertrial interval of 1, 3, 5 or 9 s with short jitters being overrepresented. Within a block, the presentation order of trials was randomized and counterbalanced with jitters across trial types. E-Prime software (Psychology Software Tools, Pittsburgh, PA) was used to present stimuli and record response accuracy and reaction time (RT). The behavioural Stroop effect was calculated by determining the difference in RT between incongruent and congruent trials, *Z*-scored (zRT) relative to all trials within an individual to control for baseline RT differences. This approach controls for mean differences in RT across individuals to isolate the relative impact of the conflict within an individual.^56-59^ Linear regression controlling for age tested whether individuals who later developed dementia differed in zRT or accuracy compared to those that remained cognitively stable.

fMRI data were acquired on a Siemens Trio 3T scanner. T_1_-weighted images were acquired using a magnetization-prepared rapid gradient-echo sequence with repetition time = 2400 ms, echo time = 3.16 ms, flip angle = 8°, field of view = 256 mm, in-plane resolution = 176 × 256 mm and slice thickness = 1 mm acquired in sagittal orientation. Images had a 1-mm isotropic resolution. BOLD data were acquired using a T_2_*-weighted gradient-echo interleaved echo-planar imaging sequence with repetition time = 2000ms, echo time = 25 ms, flip angle = 90°, field of view = 256 mm, in-plane resolution = 64 × 64 mm and slice thickness = 4 mm. Thirty-six sagittal slices were acquired parallel to the anterior-posterior commissural line. Images had a 4-mm isotropic resolution.

### Functional analyses

Pre-processing and subject level analyses followed previously published methods.^45^ Briefly, data processing included field inhomogeneities correction, high-pass filtering with a 150 s cut-off, and smoothing with a 5-mm full-width half-maximum Gaussian kernel. fMRI data were aligned to participants T_1_ structural image which was non-linearly aligned to an age-normative template in MNI space. Individuals where either run had framewise relative movement more than 0.5 mm or an average absolute movement greater than 1.5 mm were excluded based upon a 2.5 standard deviation cut-off from the population motion characteristics. For those subjects included in the analyses, the amount of motion did not differ between those who later went on to be demented and those that remained unimpaired for either relative (*t* = 0.25, *P* = 0.81) or absolute motion (*t* = 0.53, *P* = 0.60).

Event-related design analyses were conducted using FEAT.^60^ First-level analyses within a participant estimated the BOLD changes tied to the three different trial conditions (congruent, incongruent and neutral). Individual trials were modelled as a 1-s event convolved with a double-gamma haemodynamic response function and contrasts estimated the evoked response to each trial type as well as the differences between conditions (e.g. incongruent—congruent). The two task runs were combined within an individual using a fixed effects model. The lower-level models were aggregated across individuals into a higher-level analysis using FMRIB’s Local Analysis of Mixed Effects^61^ with participant modelled as a random effect.

We conducted three group level analyses. All analyses focused on changes in BOLD signal between incongruent and congruent trials. The (1) first analysis was a simple population average of the BOLD Stroop effect across all individuals. This analysis identified brain regions that were generally modulated by the cognitive demands of the task paradigm. Age was included as a covariate. The (2) second analysis explored the brain–behaviour relationship, that is, whether an individual’s behavioural Stroop effect predicts the degree to which activity in the brain changes. These models included including subject level zRT costs as the main predictor of interest as well as age as a covariate. Of interest was whether increasing behavioural costs were associated with an increased or decreased change in the BOLD signal. Finally, the (3) third analysis considered longitudinal cognitive status. This analysis compared the size of the BOLD Stroop effect between those who later became impaired (CDR > 0, *n* = 15) to those who remained cognitively normal (CDR = 0, *n* = 94). In addition to the group factor, age was included as a covariate.

Voxel-wise significance for all analyses was determined using default settings in FEAT, including an initial z-threshold of 2.3 to identify candidate voxels and then a family-wise error rate corrected cluster level significance of *P* = 0.05. All statistical maps for the functional analyses are visualized using the Python program Brainspace. For visualization of the underlying data, group level significant maps were used as a mask and underlying z-stat data was extracted using Featquery. This was done for transparency to display the distribution of the underlying data resulting in the significant voxel-wise effect. *Post hoc* analyses related the extracted effects that discriminated between groups (stable versus impaired) against a neuropsychological composite.

## Results

### Individuals who become cognitively impaired have elevated Alzheimer’s disease risk

Individuals underwent annual follow-up clinical assessments for an average of 6.8 ± 1.9 years after their baseline scans. *χ*² tests indicated that those who became impaired were more likely to have an *APOE* ε4 allele (*χ*^2^ = 4.81, *P* = 0.03) and be Aβ+ (*χ*^2^ = 26.71, *P* = 0.000002), but there was no significant difference in sex distribution (*χ*^2^ = 0.65, *P* = 0.42). Those that later progressed were older than those that remained unimpaired (mean 72.4 versus 63.5, *t* = 5.60, *P* = 0.00001). Controlling for age, those who progressed had higher global SUVR values (t = 6.84, *P* = 0.000000005) but showed no significant differences in baseline cognitive measures including MMSE (*t* = 0.06, *P* = 0.95), Knight PACC (*t* = −0.99, *P* = 0.32), Stroop zRT (*t* = 0.94, *P* = 0.35) or overall accuracy on the Stroop task (*t* = 0.23, *P* = 0.82). There were also no significant effects when looking at the raw RTs for or congruent trials (*t* = −0.55, *P* = 0.58), neutral trials (*t* = −1.17, *P* = 0.25), incongruent trials (*t* = −1.26, *P* = 0.21) or for the overall the raw Stroop RT effect (*t* = −1.94, *P* = 0.06). The average lag between MRI session and PIB Pet session was 63.9 days. In regression models controlling for age, this lag did not significantly relate to the zRT effect (*t* = −0.29, *P* = 0.79) or differ between those who progressed and remained stable (*t* = 1.73, *P* = 0.09).

### Brain activity in dorsal attention and lateral prefrontal regions increases in incongruent trials

**Figure 1:**
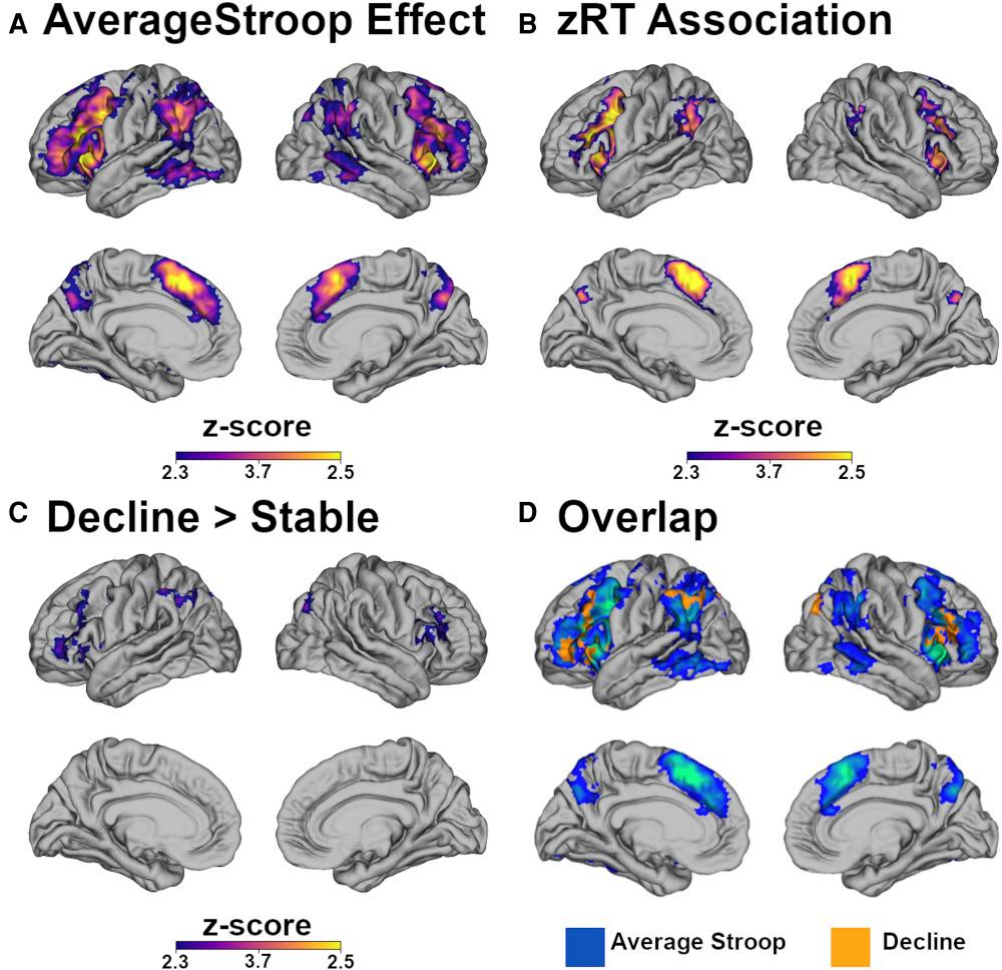
**Brain activity maps.**  *Z*-score maps representing areas of increased signal change to incongruent relative to congruent trials. The (**A**) average effect across all individuals (*n* = 109), (**B**) areas with increased modulation relative to increased behavioural zRT costs, (**C**) areas with increased modulation in those that later developed dementia (*n* = 15) relative to participants who remained cognitively normal (*n* = 94), and the (**D**) overlap between **A** and **C**. Statistical values for individual clusters are presented in Table 2.

Group level analyses revealed areas where the BOLD signal increased on incongruent relative to congruent trials (Fig. 1A). Areas of increased activity included a number of parietal and frontal networks commonly seen upregulated with attentionally demanding tasks (Table 1). Unsurprisingly, these results mirror prior analyses of a subset of these data examining trial level Stroop effects.^45^ No regions showed significantly greater activity on congruent trials relative to incongruent trials.

**Table 1:** Demographics (TBD)

	Stable (*n* = 94)	Progressors (*n* = 15)	Stat	*P*-value
Age mean (SD)	63.5 (5.4)	72.4 (7.2)	*t* = −5.60	*P* = 0.00001
Sex, F	55/94 (58.5%)	11/15 (73.3%)	*χ* ^2^ = 0.65	*P* = 0.42
APOE ε4	26/94 (27.7%)	9/15 (60%)	*χ* ^2^ = 4.81	*P* = 0.03
Ethnicity White	89/94 (95%)	13/15 (87%)	*χ* ^2^ = 0.37	*P* = 0.54
Aβ+	14/94 (14.9%)	12/15 (80%)	*χ* ^2^ = 26.71	*P* = 0.000002
PiB SUVR ^a^ (SD)	1.23 (0.47)	2.48 (0.88)	*t* = 6.84	*P* = 0.000000005
Centiloids ^b^ (SD)	7.9 (21.3)	64.0 (39.7)		
MMSE ^a^ (SD)	29.3 (1.1)	29.2 (1.3)	*t* = 0.06	*P* = 0.95
PACC ^a^ (SD)	0.18 (0.43)	0.06(0.39)	*t* = −0.99	*P* = 0.32
zRT ^a^ (SD)	0.62 (0.30)	0.74 (0.23)	*t* = 0.94	*P* = 0.35
Accuracy ^a^ (SD)	0.98 (0.02)	0.99 (0.02)	*t* = 0.23	*P* = 0.82

^a^ *t*-stat is from models including age as a covariate.

^b^Centiloids are a linear transformation of SUVRs.

### Increased activity correlates with greater Stroop effects

**Figure 2:**
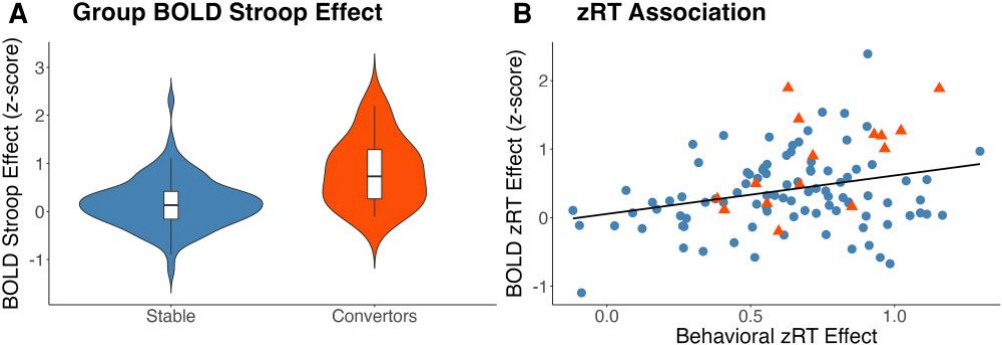
**Brain and behaviour relationships.**  *Z*-score values extracted from the voxel-wise contrasts presented in Fig. 1 which are looking at change in the BOLD signal for incongruent relative to congruent trials to illustrate the underlying data distribution driving the statistical results. As the masked used to extract the data are derived from statistically significant voxels, these plots are for illustrative purposes and no new statistics are being performed. (**A**) A violin and box plots showing differences between those that later developed dementia (CDR > 0, *n* = 15 orange) relative to participants who remained cognitively normal (CDR = 0, *n* = 94, blue). This is the data extracted from the significant voxels in Fig. 1C. (**B**) A scatter plot showing the association between behavioural zRT costs and the fMRI Stroop effect in the entire cohort (*n* = 109). This is the extracted data from the significant voxels in Fig. 1B. For visualization, orange dots represent those participants that became impaired (*n* = 15) and blue dots represent those participants who remained cognitively unimpaired (*n* = 94).

The second set of analyses examined the relationship between regional activation and zRT differences between incongruent and congruent trials. There was a significant, positive relationship such that larger behavioural Stroop effects were associated with greater task activation, particularly in dorsal attention and lateral prefrontal regions (Fig. 1B). The voxels that were positively associated with the zRT effect were largely within the omnibus mask (99.4%). There were no clusters that demonstrated a significant negative association with the behavioural zRT Stroop predictor. For visualization purposes, Fig. 2B plots behavioural zRT Stroop effects against the subject level fMRI Stroop effect in the significant voxels. Since accuracy was near ceiling (∼99%), analyses only considered RT.

### Activity at baseline predicts clinical conversion

The third contrast found that individuals who later progressed to dementia demonstrated a greater BOLD Stroop effect (Fig. 1C) than their peers who remained cognitively normal. The voxels that discriminated between cognitively stable and progressor groups overlapped (92.4%) with the omnibus maps generated in the whole cohort. There were no regions where individuals who remained cognitively normal demonstrated greater activity. For visualization purposes, Fig. 2A has a violin plot split by group of the fMRI Stroop effect in these significant voxels.

**Figure 3:**
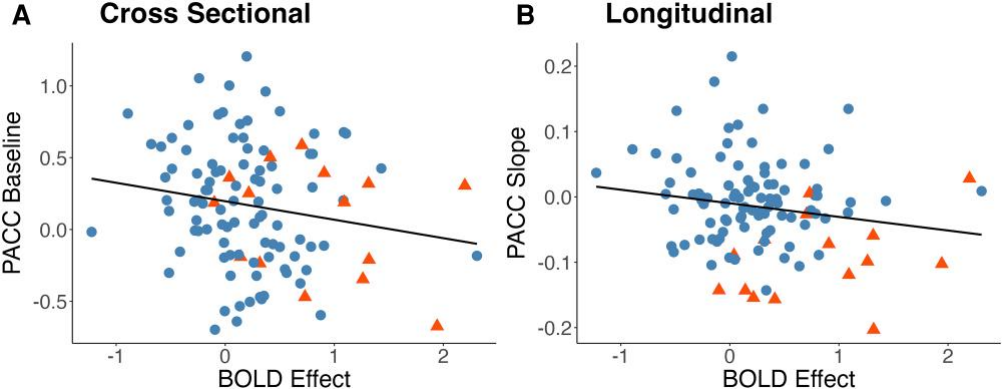
**Relationship with neuropsychological scores.** Associations in all participants (*n* = 109) between the extracted BOLD Stroop effect (z-score) in the significant voxels discriminating participants who remained stable and those who developed dementia (Fig. 1C) against a neuropsychological composite (*Z*-score). Each point in the graph represents one individual in the study with the colours and shapes indicating individuals who remained unimpaired (*n* = 94, blue) and those who became impaired (*n* = 15, orange). The plot depicts the association between the BOLD Stroop effect and (**A**) cross-sectional and (**B**) longitudinal neuropsychological performance. The *x*-axis is the extracted *Z*-score BOLD effect while the *y*-axis reflects the baseline cognitive performance or change in performance, respectively. Cross-sectional associations were fit using linear regressions while also controlling for age, and there was a non-significant (*t* = −1.87, *P* = 0.07) association in the regression model with the BOLD signal predicting baseline neuropsychological performance. Longitudinal neuropsychological data were fit using linear mixed effects models while also controlling for age with the main predictor of interest being how well the extracted BOLD predicted change in neuropsychological performance. There was a non-significant regression term (*t* = −1.25, *P* = 0.21) for baseline BOLD modulation predicting longitudinal change in neuropsychological performance.

Rather than examining cognition dichotomously, *post hoc* tests examined the relationship between baseline fMRI activity in the regions sensitive to progression (Fig. 1C) and performance on a neuropsychological composite (Fig. 3). When examining the relationship between the BOLD change and baseline PACC, there was a non-significant association (*t* = −1.87, *P* = 0.07) such that and increased BOLD Stroop effect was related to worse cognitive performance. This association remained unchanged when including baseline age in the model (*t* = −1.85, *P* = 0.07) as age was not associated with PACC performance (*t* = −0.09, *P* = 0.93). Associations between baseline BOLD effects and longitudinal cognition were done using linear mixed effects models predicting Knight PACC scores as a function of time while at the participant level including both random slopes for the time predictor and intercepts in the model. There was a non-significant association with the Stroop BOLD effect at baseline predicting more rapid cognitive decline on the PACC (*t* = −1.25, *P* = 0.21). This association was relatively unchanged (*t* = −1.35, *P* = 0.18) when including the main effect of age and the interaction of age and time (*t* = −3.45, *P* = 0.006) as predictors in the model.

## Discussion

The present study aimed to investigate the significance of altered task-evoked brain activity in older adults. While increased activity with ageing is commonly observed in the literature,^3-19^ the degree to which such activity is detrimental is debated. Using a longitudinal design, we related baseline brain activity to longitudinal cognitive outcomes. We found that individuals who later develop dementia exhibited greater baseline brain activity in response to attentionally demanding cognitive tasks, even though no differences in cognition were observed at baseline. This strongly argues that although heightened activation of attentional control networks may be beneficial in the short term, overactivation of attentional control networks predict worse long-term cognitive prognosis. Our findings are consistent with multiple theories of cognitive ageing^33,34^ and address a critical gap in the literature as prior work has overwhelmingly used only cross-sectional associations between brain activity and cognition. These data show what is typically attributed to ageing may in fact reflect subtle disease-related changes detectable years before the onset of overt dementia.

Our results reveal three main findings. First, we identified a canonical set of networks activated during incongruent trials, in which the colour word conflicts with its font colour, compared to congruent trials. The Stroop colour-naming task is a cognitive test that requires attentional control to accurately name colours on incongruent trials. The discrepancy between the colour word and its font colour experienced during Stroop conflict imposes a cognitive demand which demands selective attention and inhibition. We find prominent areas of activation in the anterior cingulate cortex (ACC), dorsolateral prefrontal cortex (DLPFC) and posterior temporal regions (Fig. 1 and Table 2). These patterns of results are highly consistent with prior work examining the Stroop paradigm using fMRI.^26,62-66^ Some of the regions overlap with the DMN, indicating a modest degree of reduced suppression of these areas.

**Table 2:** Descriptive information for significant clusters

Contrast	Cluster	Label	Voxels	*P*	Z-Max	COG X	COG Y	COG Z
Average	7	Anterior cingulate	15 758	<0.001	7	−5	22	25
	6	Angular gyrus (L)	7444	<0.001	5	−37	−58	32
	5	Angular gyrus (R)	2084	<0.001	4	45	−54	40
	4	Cerebellum	1199	<0.001	4	2	−71	−26
	3	Thalamus (R)	898	<0.001	4	13	−6	4
	2	Middle temporal gyrus (R)	700	<0.01	4	59	−42	−2
	1	Thalamus (L)	583	0.01	4	−12	−6	5
zRT	6	Inferior frontal gyrus (L)	2860	<0.001	7	−42	18	21
	5	Angular gyrus (L)	1862	<0.001	5	−42	−55	41
	4	Inferior frontal gyrus (R)	1632	<0.001	5	43	19	15
	3	Superior frontal gyrus	1595	<0.001	6	0	16	52
	2	Angular gyrus (R)	308	<0.001	4	47	−49	41
	1	Precuneus	192	<0.01	5	2	−70	41
Decline	5	Lateral occipital cortex superior (L)	1036	<0.001	4	−33	−62	41
	4	Inferior frontal gyrus (L)	900	<0.001	3	−43	28	15
	3	Lateral occipital cortex superior (R)	521	0.02	4	34	−73	36
	2	Inferior frontal gyrus (R)	481	0.03	3	48	22	21
	1	Cerebellum	450	0.04	4	−2	−79	−28

Labels were derived from the Harvard-Oxford Atlas and indicate the region with the high probability for the given coordinate. COG = centre of gravity which represents the centroid of the cluster. Z-max is the maximum *Z*-score found within each cluster and voxels represent the number of voxels in each cluster. Cluster data is reported for the overall group average BOLD difference between incongruent and congruent trials, the association between BOLD Stroop effect and individual’s behavioural effect, as well as the group difference in the Stroop effect between those who later become impaired and those who remained cognitive normal.

Second, we find that network activity reflects the degree of behavioural conflict an individual experiences at the trial level. The utilization of zRT means observed effects represent the subject-specific behavioural cost rather than other features such as age-related generalized slowing. Most prior studies on age-related increases in functional activity do not account for individual-level behavioural associations. While group level findings indicate that more attentionally demanding tasks lead to increased activity,^31,36-38^ individual-level predictions are less clear. In the current study, across individuals greater relative behavioural Stroop effects correlated with increased activity in frontal regions, particularly the ACC and DLPFC (Fig. 1 and Table 2). This suggests that as levels of internal conflict rise, there is greater activation of frontal control networks, likely due to active recruitment of these regions. Alternatively, a negative association could have emerged, indicating that individuals with the greatest activations have mobilized the most resources, resulting in lower behavioural cost of semantic conflict. It would also have been plausible to observe a negative correlation under the scaffolding theory of ageing^33,34^ where both activity and behaviour collapse when task demands exceed the capacity for the compensatory recruitment of resources. Given that overall accuracy on the Stroop task was very high (mean = 98.4%, standard deviation = 1.8%), this did not occur. This individual-level association provides greater context for interpreting the longitudinal results of our study.

Finally, we found that greater recruitment of cortical areas during attentionally demanding conditions corresponded with an increased risk of developing dementia. This difference in brain activity between groups was present at baseline, even in the absence of overt behavioural differences in accuracy, RT or on a neuropsychological composite. The regions most strongly associated were the DLPFC and lateral parietal cortex (Fig. 1 and Table 2). Theories of cognitive ageing suggest that increased brain activity is not a monolithic process, but a dynamic one. Greater activation of task-associated networks or the recruitment of additional networks is an adaptive process to meet ongoing cognitive demands. Such patterns are most likely to occur in older adults^3-10^ but can also occur in younger adults given the right conditions.^31,36-38^ However, as cognitive demands continue to rise, the capacity to recruit additional resources eventually diminishes, leading to cognitive decline. Our findings show that otherwise cognitively normal individuals who exhibit elevated activity at baseline are more likely to develop dementia over time. This suggests that while employing compensatory mechanisms may initially be successful, the very need to do so indicates subtle declines which progress and ultimately lead to dementia.

*Post hoc* analyses examined the evoked activity on the Stroop task that predicted later dementia and a neuropsychological composite. Both cross-sectionally and longitudinally, there was an association such that greater evoked activity predicted worse cognition (Fig. 3), although the strength of these relationships did not reach significance. These results are consistent with the results showing that task activity in these regions is sensitive to cognitive change as measured by dementia status. The fact that these associations did not reach statistical significance could be due to a couple factors. First, the transition to overt dementia is focused on cognition that is deemed pathological. This categorization, rather than examining continuous cognition, may simply be providing slightly more statistical power. Second, neuropsychological tests can be susceptible to learning effects with repeated testing partially masking longitudinal decline. Future work with larger samples should investigate the subtleties of the relationships between continuous and categorical cognitive measures.

Given the dearth of ageing studies relating fMRI to longitudinal cognitive outcomes, our work is important for validating proposed theoretical models. Although our results align with established theories of cognitive ageing, these findings also raise questions about how ‘healthy ageing’ needs to be defined. Alzheimer's disease is the most common age-related neurodegenerative disease, and its pathology evolves silently over the course of decades, resulting in a long preclinical stage before the onset of dementia symptoms.^67,68^ Post-mortem studies demonstrate that a substantial proportion (30–40%) of non-demented older adults meet neuropathological criteria for an Alzheimer's disease diagnosis.^69^ Similar observations have been made using in vivo PET imaging.^70-74^ As the frequency of preclinical Alzheimer's disease increases as a function of age, it may confound what we interpret as ‘healthy’ ageing. Prior work has shown that non-demented individuals with Alzheimer's disease pathology exhibit worse cross-sectional and longitudinal performance on neuropsychological tests.^75-78^ Further, age-related declines in cognition have been overestimated by the unintentional inclusion of individuals with preclinical Alzheimer's disease.^39,40^ In our study, participants who later became impaired had high levels of Aβ at baseline, with 80% classified as Aβ+ (12/15) and 60% as APOE ε4 carriers (9/15). This strongly suggests that our results are largely driven by preclinical Alzheimer's disease.

Although a predominant feature of Alzheimer's disease is profound memory loss, a substantial body of literature indicates that changes in attentional control occur in both impaired individuals and during the preclinical phase.^79-83^ We, as well as others, have also shown reduced deactivations of the DMN^84-88^ and increased activity in attentional networks^35,45,88-91^ in cognitively normal individuals at risk for developing Alzheimer's disease due to carrying an ε4 allele or having abnormal levels of preclinical pathology (see McDonough *et al*.^92^ for meta-analysis). Thus, Alzheimer's disease pathology could play a role in shaping our understanding of pathological ageing well beyond tests of memory. Given that the vast majority of studies of ageing do not include biomarker testing, it is highly likely we as a field are conflating patterns of ‘ageing’ with early manifestations of disease. Conversely for individuals focused on neurodegenerative disorders, the current work also demonstrates that functional activity in the brain changes well in advance of over dementia onset. Altered fMRI activity has also been shown to predict progressive decline even in individuals who are already impaired.^93^ Understanding how these changes in the brain play out can provide insights into the preclinical phase of the disease which will likely become the target of future therapeutic interventions. The rise of cheap and easily accessible blood-based biomarkers of Alzheimer's disease pathology^94-96^ could prove a boon to both fields as such testing becomes seamlessly integrated into more studies of ageing.

The current work provides important insights into understanding task-evoked brain activation changes, but it is not without limitations. With over 100 individuals, the sample of individuals with longitudinal cognitive assessment is robust and comparable to other works noted in the introduction and discussion. However, even with this sample, only a modest number of individuals became impaired (*n* = 15). Future studies with larger populations are needed to replicate the current work and to better understand the relationship between baseline activity and longitudinal cognition. Similarly, while having an average follow-up of ∼7 years is powerful, a greater duration of study would provide even more nuanced insights into the effects of preclinical Alzheimer's disease on the brain. Some individuals who remained cognitive normal did so despite having abnormal levels of amyloid at baseline. With a longer duration of follow-up, an even greater proportion of individuals would likely decline. A greater duration of follow-up, and more frequent testing with task fMRI, would provide insight into how proximal a risk factor overactivation represents. Further, the observed effects are relatively modest in their effect size (Fig. 2 and Table 2) and the relative functional changes during the preclinical phase are likely to be subtle, rather than a dramatic, effects. Also, while the current models control for age, there are also unmeasured factors (e.g. blood flow, neurovascular coupling, time on task, etc.) that could differ between those who later become impaired and those who remain stable that could contribute the observed functional differences.

Given the scarcity of studies relating task activity to longitudinal cognition, more convergent studies and ideally meta-analyses are needed to assess the certainty of the current results. The current psychological paradigm is also one that heavily engages attentional control. Studies of ageing and Alzheimer's diease utilize a mixture of paradigms that engaged to varying degrees attentional control as well as memory but rarely systematically tests both systems. As attention and memory are strongly linked, a more nuanced understanding of functional and cognitive changes would emerge with studies explicitly multiple domains. Finally, those individuals who became demented overwhelmingly represented an Alzheimer's disease phenotype. It is also of interest to know whether the findings presented here would generalize to other causes of impairment (e.g. vascular dementia) or what role, if any, other age-related co-pathologies play.

Further, a key feature of several models of cognitive ageing revolves around U-shaped functions that can only be revealed by testing individuals with different levels of difficulty. Paradigms with multiple levels of difficulty could have produced a different pattern of results than observed here. For example, based upon the prior literature, we could predict that individuals that later become impaired may show greater activation at lower difficulty levels but reduced task-evoked activity at greater levels of difficulty. Future work with larger cohorts, greater follow-up and a more diverse array of tests could further enhance our understanding of cognitive ageing.

Prior work has also noted that both increased and decreased connectivity in resting-state fMRI which can predict future cognitive decline.^97,98^ Although not a direct parallel to task-evoked paradigms with tap into specific cognitive systems, resting-state analyses may provide important insights into brain integrity. Resting-state fMRI provides support for a cascading network failure model^99,100^ as pathology progressive spreads throughout the brain. Such models are complimentary to cognitive ageing models such as scaffolding in that the eventual failure of compensatory mechanisms likely corresponds with progression to new brain areas in the cascading network models. The continued failure of compensatory mechanisms, and further network declines, likely occurs as dementia worsens in later dementia stages (see Corriveau-Lecavalier *et al*.^101^).

Overall, this work retrospectively analyses differences in brain activity in cognitively normal individuals based on follow-up clinical evaluations spanning up to 7 years. Even in the absence of overt behavioural differences, a greater recruitment of functional brain networks at baseline predicted an increased risk of later developing dementia. Thus, the attentional mechanisms postulated to drive functional changes in ageing may in fact be transiently compensatory and may also herald the underlying progression of undiagnosed neurodegenerative processes.

## Data Availability

All data included in these analyses are available from the Charles F. and Joanne Knight Alzheimer's Disease Reserach Center through an online portal https://knightadrc.wustl.edu/data-request-form/. This manuscript is the result of funding in whole or in part by the National Institutes of Health (NIH). It is subject to the NIH Public Access Policy. Through acceptance of this federal funding, NIH has been given a right to make this manuscript publicly available in PubMed Central upon the Official Date of Publication, as defined by NIH.
